# Democratizing Specialized Care in the Digital Age: Project ECHO as a Learning Environment for Continuing Professional Development

**DOI:** 10.3390/healthcare14070824

**Published:** 2026-03-24

**Authors:** Ilian Cruz-Panesso, Lucie Fuzeau, Brenda Lécuyer, Mélanie Demers

**Affiliations:** Direction de l’Enseignement et de l’Académie (DEAC), Centre Hospitalier de l’Université de Montréal (CHUM), Montreal, QC H2X 0A9, Canada; lucie.fuzeau.chum@ssss.gouv.qc.ca (L.F.); brenda.lecuyer.chum@ssss.gouv.qc.ca (B.L.); melanie.demers.chum@ssss.gouv.qc.ca (M.D.)

**Keywords:** Project ECHO, telementoring, continuing professional development, digital health education, learning architecture, learning sciences, clinical reasoning, assessment alignment

## Abstract

Background: Digital health technologies have reshaped continuing professional development (CPD) in healthcare. However, learning in digitally mediated programs is often assumed rather than explicitly designed and assessed. Project ECHO^®^ (Extension for Community Healthcare Outcomes), a globally implemented telementoring model, expands access to specialized expertise through videoconferencing-based, case-oriented learning. While prior literature has documented program reach, implementation, and clinical outcomes, comparatively less attention has been paid to the interactional mechanisms through which learning unfolds within ECHO sessions. Objectives: This article conceptualizes Project ECHO as a structured learning environment and proposes a theoretically grounded framework for examining and assessing learning processes in digital CPD. Methods: Using situated learning, communities of practice, and cognitive apprenticeship as analytical lenses, this conceptual analysis examines participation structures, distributed expertise, facilitation practices, and case-based dialogue in ECHO sessions. Principles of constructive alignment inform a process-oriented assessment approach aligned with CPD evaluation models such as Moore’s framework. Conceptual framework: This article develops a theory-informed framework that conceptualizes Project ECHO as a structured learning architecture for digital continuing professional development. The framework identifies how participation, distributed expertise, facilitation, and case-based dialogue support learning processes during ECHO sessions. It also proposes process-oriented indicators to make learning dynamics more visible alongside outcome-based evaluation approaches. Conclusions: By foregrounding learning processes, this analysis offers a conceptual foundation to strengthening pedagogical alignment, faculty development, and assessment design in ECHO programs. The framework contributes to digital CPD scholarship by clarifying how learning develops within telementoring environments and by guiding future research and program refinement. More specifically, the article contributes a process-oriented evaluation perspective that helps make learning quality more visible within telementoring environments, thereby complementing dominant outcome-focused CPD models.

## 1. Introduction

Digital transformation reshapes healthcare not only in the delivery of care but also in how health professionals access knowledge, collaborate, and engage in continuing professional development (CPD). This article examines Project ECHO^®^ (Extension for Community Healthcare Outcomes) as a learning environment for CPD and explores how videoconferencing-based telementoring structures and supports learning. Rather than treating learning as an implicit outcome of participation, this conceptual paper adopts a learning sciences perspective to examine the educational mechanisms operating within the ECHO model and to inform the development of a conceptual framework for understanding how learning processes unfold within ECHO sessions.

Health systems increasingly rely on videoconferencing-based education and digital knowledge translation platforms to address rising clinical complexity and persistent inequities in access to specialized expertise, particularly in geographically dispersed or underserved contexts [1,2]. Within this landscape, ECHO has become one of the most widely implemented telementoring models. The University of New Mexico School of Medicine developed the model in 2003 to improve access to hepatitis C care in underserved and correctional settings. The model connects community-based clinicians with multidisciplinary specialist teams through regularly scheduled, case-based videoconferencing sessions [3].

Much of the existing literature on ECHO has focused on organizational reach, scalability, and reported clinical and service-level outcomes. Reviews document the expansion of ECHO programs across clinical domains and geographic regions and report indicators such as program volume, participation rates, increased access to specialist expertise in underserved settings, and clinical outcomes associated with program implementation [4,5,6,7]. Although these findings remain relevant, they offer limited insight into how learning unfolds during ECHO sessions. Despite the centrality of the “all teach, all learn” principle within the ECHO model, learning is often treated as an implicit outcome of participation rather than as a process shaped by pedagogical design. As a result, important questions remain regarding how telementoring environments support interaction, clinical reasoning, and reflective dialogue among participants.

A growing body of work has begun to explore the educational dimensions of ECHO programs by documenting case-based dialogue, knowledge exchange, and perceived gains in professional confidence and competence among participants [8,9,10,11]. These studies provide important indications of learning-related outcomes. However, comparatively less attention has been given to pedagogical mechanisms through which learning interactions are organized, facilitated, and sustained within ECHO sessions. As a result, questions remain regarding how telementoring environments support interaction, clinical reasoning, and collective reflection in ways that contribute to professional learning.

Relatively little work has examined Project ECHO through learning-theory perspectives. One notable example is the study by Socolovsky et al. [8], which demonstrates that participants’ experiences in a pilot ECHO curriculum reflected elements of social cognitive theory, situated learning, and communities of practice, providing early theoretical grounding for the model’s educational potential. Building on this foundation, the present article moves beyond identifying theoretical alignment to develop a more explicit conceptual framework that clarifies how learning processes are structured within ECHO environments.

Specifically, this article proposes a theory-informed framework that conceptualize Project ECHO as a structured learning architecture for digital continuing professional development. Drawing on insights from situated learning, communities of practice, and cognitive apprenticeship, the framework identifies the interactional mechanisms through which learning unfolds during ECHO sessions. It also introduces a set of process-oriented indicators designed to make learning dynamics more visible during telementoring exchanges. In doing so, the analysis complements outcome-focused CPD evaluation approaches, such as Moore’s framework by clarifying how learning processes could potentially emerge within the educational interactions that characterize ECHO programs.

## 2. Telementoring as a Learning Modality in Digital CPD

Telementoring—also referred to as e-mentoring or online mentoring—describes mentoring and learning relationships mediated through digital educational networks rather than face-to-face interactions [12]. In healthcare CPD, telementoring initiatives such as Project ECHO use these networks to connect geographically dispersed clinicians, support interprofessional dialogue, and situate learning within ongoing clinical practice [2].

Approaching telementoring as a learning modality requires more than describing its technological infrastructure. Learning does not arise from connectivity alone, but from deliberate alignment between educational intentions, learning activities, facilitation strategies, and evaluation approaches [13]. In CPD initiatives delivered through digital educational networks, design decisions—such as case selection, participant roles, interaction formats, and facilitation practices—therefore play a central role in shaping both participants’ learning experiences and the dimensions of learning that can be meaningfully observed, supported, and assessed.

Evaluation frameworks commonly used in CPD, such as Moore’s outcomes framework for continuing medical education, structure the assessment of educational initiatives across sequential levels of impact [14,15]. The model conceptualizes outcomes from participation and satisfaction, through learning and competence, to changes in performance and, in some cases, patient and community health outcomes [5,7]. Although this approach supports accountability and comparability across programs, it primarily captures outcomes that occur after participation and offers limited leverage for examining learning as it unfolds during telementoring interactions.

More recent work in CPD and instructional design emphasizes the importance of aligning evaluation approaches with instructional design principles and learning processes when interpreting educational impact [13,16]. These arguments stress the need for coherence between what the program aims to achieve, how learning is structured, and how impact is assessed. From a pedagogical standpoint, this logic aligns with principles of constructive alignment, which emphasize coherence between intended learning outcomes, learning activities, and assessment approaches, with learning theories guiding these design decisions from the outset [17,18].

For telementoring initiatives that aim to function as learning environments, alignment must therefore begin at the design stage. Learning theories provide a foundation for articulating educational objectives, selecting and structuring learning activities, and choosing evaluation strategies that attend to learning processes rather than only to distal outcomes. When programs articulate this alignment from the outset, evaluation can focus on how participants engage with cases, how reasoning develops through interaction, and how facilitation supports collective sense-making over time.

While outcome frameworks such as Moore’s support accountability in CPD [14], the analysis presented in this article complements them by examining the pedagogical processes that learning theories identify as central to professional learning in digital educational networks.

## 3. Conceptual Analytical Approach

This article adopts a conceptual analytical approach in which established learning theories are used as interpretative lenses to examine how Program ECHO structures learning interactions and supports knowledge construction. Rather than testing predefined hypotheses, the analysis uses these frameworks to clarify key pedagogical processes and to develop theoretically grounded constructs for understanding how learning unfolds within ECHO sessions. In this approach, learning theories guide the analysis of learning interactions by helping identify key mechanisms that shape participation, reasoning, and knowledge exchange within the ECHO environment. They also support the development of theoretically grounded constructs that explain how learning processes unfold in practice [19,20]. Such theory-guided analysis is used in theory-building research, where existing conceptual frameworks provide the basis for interpreting complex organizational and learning processes and for generating explanatory models of practice [21,22].

In the present analysis, three perspectives were used to guide interpretation of learning processes within Project ECHO: situated learning, community of practice, and cognitive apprenticeship. These three perspectives were selected because, together, they address key aspects of learning within ECHO programs. Situated learning emphasizes participation in authentic clinical practice. Communities of practice highlight learning through social interaction and the development of shared repertoires among participants. Cognitive apprenticeship focuses on how expert reasoning becomes explicit through guided discussion. Although other theoretical perspectives could also inform the analysis, these frameworks were selected because they align closely with the case-based, interactive, and practice-oriented structure of ECHO sessions.

The analysis is conceptual in nature and is grounded in established descriptions of ECHO program design and session structure. Learning theories provide the analytical lens for conceptualizing how case-based discussion may structure participation, how facilitation may support the articulation of clinical reasoning, and how engagement with uncertainty can be interpreted as part of the pedagogical dynamics of ECHO sessions.

The theoretical interpretation developed in this article draws on three sources: published descriptions of the ECHO model, literature documenting session structure and facilitation practices, and institutional reflection arising from the Quebec experience as a practice-based reference point. These sources were not treated as empirical data for analysis but as conceptual material used to identify recurring design features relevant to theory-guided interpretation.

Building on this interpretation, theoretical constructs derived from the selected learning frameworks were translated into illustrative indicators that can guide the observation and assessment of learning processes in telementoring contexts, as summarized in the tables presented in this article. These indicators and associated assessment approaches are proposed as conceptual guides grounded in theoretical framework, rather than as validated measurement. This perspective maintains coherence between educational objectives, learning activities, and analytical focus, enabling a process-oriented examination of learning without framing the analysis as an empirical validation or reducing learning to outcome-based metrics alone.

In this article, the experience of the Centre Hospitalier de l’Université de Montréal (CHUM), a large academic teaching hospital in Quebec, Canada, is used as an illustrative institutional context that informs the pedagogical interpretation of Project ECHO’s design and sustainability. Rather than presenting CHUM as an empirical case study, the discussion draws on this context to situate the proposed framework within a real organizational environment.

Table 1 and Table 2 further develop this theoretical interpretation by translating the selected theories into learning constructs and illustrative indicators. These elements provide concrete reference points that can guide the observation and assessment of learning processes as they unfold during ECHO discussions.

## 4. A Learning Architecture for Project ECHO

Project ECHO builds on inspiration from apprenticeship-based learning practices long embedded in medical training, particularly learning through case discussion and guided clinical reasoning during clinical rounds [23,24]. The model organizes learning through a recurring configuration that combines videoconferencing sessions, participant presented cases, and facilitation by an interprofessional expert hub. This configuration establishes a stable learning architecture anchored in real clinical practice.

In this article, learning architecture refers to the way an educational environment is deliberately structured to support learning over time. In the context of Project ECHO, it includes how participants interact, how cases are discussed, how facilitators guide the dialogue, and how these elements are organized across recurring sessions to sustain collaborative clinical reasoning.

A core architectural principle of ECHO is “all teach, all learn.” When a community-based clinician presents a case marked by diagnostic uncertainty, the discussion unfolds as a collaborative exchange. Specialists and peers ask clarifying questions, offer alternative interpretations of the clinical situation, and consider possible treatment strategies while taking into account the realities of the presenter’s local practice context. Through this process, participants develop understanding through collective discussions rather than receiving information through a one-directional transmission of expertise.

In this environment, expertise circulates among participants instead of remaining concentrated in specialists. Community-based clinicians contribute practice-based knowledge grounded in their daily clinical contexts, while expert facilitators guide the discussion, make clinical reasoning visible, and help participants connect different perspectives during the exchange [2].

Across successive meetings, recurring case discussions and stable participation patterns gradually structure the learning environment. Regular interaction, clearly defined roles, and shared clinical problems support an ongoing process of collaborative problem-solving and professional learning. Over time, these elements form a recognizable learning architecture that sustains knowledge exchange within the ECHO network.

### Learning Processes Guiding “All Teach, All Learn” Within the ECHO Architecture

**Figure 1:**
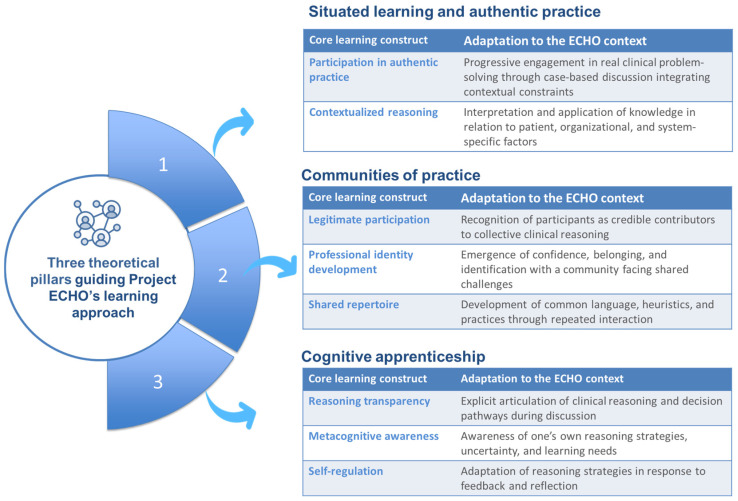
Theoretical foundations of Project ECHO as a learning architecture. The figure illustrates the three theoretical pillars underpinning Project ECHO’s learning architecture: situated learning and authentic practice [26], communities of practice [27] and cognitive apprenticeship [28]. For each pillar, we propose the core learning construct and their adaptation to the ECHO context.

Building on learning-theory perspectives, this analysis proposes that the learning architecture created by this recurring configuration—case-based sessions, distributed expertise, and facilitated dialogue—supports professional learning when its design aligns with learning processes identified by established learning theories [8]. Situated learning, communities of practice, and cognitive apprenticeship each look at complementary learning processes that emphasize learning as participation in practice, learning as social engagement within a professional community, and learning as guided development of expert reasoning [8]. In the context of ECHO, these perspectives help explain how learning emerges through engagement with authentic cases, interaction with peers and experts, and explicit articulation of clinical reasoning. The theoretical perspectives and associated learning constructs relevant to the ECHO context are summarized in Figure 1.

This learning architecture does not operate uniformly across all ECHO contexts. Its pedagogical potential may be weakened when sessions are highly hierarchical, when participant turnover is high, when psychological safety is limited, when facilitation remains primarily didactic, or when digital access and platform usability constrain participation. Group size, session frequency, hub composition, and local language or cultural dynamics [10,25] may also shape the extent to which legitimate participation and distributed expertise can be sustained over time.

This conceptual figure, developed by the authors, illustrates the three learning-theory pillars guiding Project ECHO’s learning approach: situated learning, communities of practice, and cognitive apprenticeship. For each theoretical perspective, core learning constructs are identified and mapped to their adaptation within the ECHO context. Together, these constructs explain how ECHO structures learning through authentic case-based participation, distributed expertise, and facilitated articulation of clinical reasoning. The figure highlights how the “all teach, all learn” principle is operationalized pedagogically through progressive engagement in real clinical problems, legitimate participation within a professional community, and guided development of reasoning, metacognitive awareness, and self-regulation over time.

From an integrative pedagogical perspective, the learning constructs outlined above clarify that “all teach, all learn” functions as a system of distributed expertise only when the learning architecture supports authentic cases, legitimate participation, and guided reasoning. When cases lack relevance, facilitation privileges content delivery, or expert voices dominate discussion, these learning processes weaken and telementoring reverts to transmissive formats.

## 5. Operationalizing Learning Processes in ECHO

When learning is understood as emerging from interaction and clinical reasoning rather than from content exposure alone, evaluation approaches must be aligned with the learning processes the architecture is designed to support. Learning constructs embedded in the ECHO architecture can be translated into observable indicators that bring participation, reasoning, and regulation into clearer analytical focus over time, as illustrated in Table 1. The learning constructs, illustrative indicators, and assessment approaches presented in Table 1, Table 2 and Table 3 are conceptual and illustrative propositions derived from the theoretical framework. They are intended to support the observation and analysis of learning processes and should not be interpreted as empirically validated instruments or standardized evaluation tools.

These assessment approaches can be implemented at different levels of methodological complexity. At a pragmatic level, low-burden approaches rely on strategies that fit naturally within the routine facilitation of sessions. Facilitators can document observations of learning interactions, participants can respond to brief structured reflection prompts, and programs can examine patterns of participation such as the frequency of case presentations, questions, and peer contributions. These approaches offer an initial view of how participants engage in collaborative clinical reasoning without requiring extensive analytic infrastructure.

Programs that seek a more detailed understanding of learning dynamics can adopt more analytically intensive methods. These approaches include coding session transcripts, conducting discourse analysis of case discussions, or tracking the evolution of specific clinical cases across multiple meetings. Such methods allow researchers and program leaders to examine how collective problem-solving unfolds over time.

Presenting multiple levels of evaluation enables programs to select strategies that align with their evaluation capacity and institutional priorities while maintaining attention to the process through which learning occurs.

Depending on program capacity, these approaches may be implemented by facilitators as part of reflective practice, by program leads for quality improvement purposes, or by researchers conducting more formal process analyses.

### 5.1. Ethical and Governance Considerations

ECHO discussions often involve authentic clinical situations derived from practice. Evaluation strategies that rely on recordings, transcripts, or detailed analysis of case discussions therefore require careful attention to ethical and governance considerations. Programs must align evaluation procedures with institutional policies governing confidentiality, the handling of patient-related information, and secure data storage. Clear communication with participants regarding recording practices, consent procedures, and the use of data for evaluation purposes helps maintain trust while supporting rigorous study of learning processes.

When recordings or transcripts are used, programs should establish clear procedures regarding participant information, consent where required, de-identification of case material, access control, and data retention. Depending on the purpose of evaluation, institutional ethics or quality-governance mechanisms may also need to be considered.

Recent advances in artificial intelligence can potentially offer new possibilities for capturing and analyzing conversational data generated during ECHO discussions. Automated transcription systems and conversational analytics can facilitate the documentation of learning interactions and support more systematic analysis of dialogue and reasoning processes. However, the use of such technologies requires particular vigilance regarding data governance. Programs should rely on secure institutional systems that comply with organizational standards for data protection and privacy, rather than commercial tools that do not guarantee appropriate control over sensitive information.

Because Project ECHO is frequently positioned as a response to inequities in access to specialist expertise, the quality of learning within these environments must also be considered in relation to digital access, platform usability, language, accessibility needs, power dynamics, and psychological safety. From the perspective of community of practice theory, these conditions may shape the possibilities for legitimate participation within the learning community. They influence who speaks, whose knowledge is recognized, and whether participation becomes genuinely legitimate rather than merely nominal [26,27,29,30].

These contextual conditions, including digital access, platform usability, language, accessibility needs, power dynamics, and psychological safety, also shape how participation, reasoning, and knowledge exchange become visible during ECHO discussions. Evaluation strategies aligned with interaction-based learning processes (as outlined in Section 5) must be implemented within robust ethical and governance frameworks, particularly when they involve capture and analysis of authentic clinical discussions.

**Table 1:** Operationalizing and evaluation of learning constructs in ECHO (illustrative indicators and assessment approaches).

Learning Construct	Illustrative Indicators	Possible Assessment Approaches
Participation in authentic practice	Increasing sophistication of case presentations; articulation of contextual constraints	Longitudinal case analysis; facilitator observation; reflective writing
Contextualized reasoning	Integration of evidence with patient and system factors; explicit acknowledgment of uncertainty	Discourse analysis; rubric-based reasoning analysis
Legitimate participation	Increased frequency and diversity of contributions; willingness to present cases	Participation tracking with qualitative interaction analysis
Professional identity development	Expressions of confidence, belonging, and responsibility toward peer learning	Reflective logs; professional identity instruments
Shared repertoire	Emergence of common terminology, heuristics, and reference points	Longitudinal discourse analysis; facilitator field notes
Reasoning transparency	Verbalization of assumptions, hypotheses, and decision criteria	Coding of session transcripts
Metacognitive awareness	Explicit reflection on uncertainty and learning needs	Guided reflective prompts
Self-regulation	Adaptation of reasoning strategies across sessions	Comparative analysis of early and later cases

**Table 2:** Aligning Pedagogical Intent and Assessment Focus in ECHO.

Pedagogical Intent	Common Assessment Focus in CPD	Process-Oriented Assessment Focus Aligned with the ECHO Architecture
Support shared learning	Attendance counts	Quality and diversity of participation
Foster clinical reasoning	Satisfaction ratings	Transparency and evolution of reasoning
Build professional confidence	Single-point self-efficacy scores	Longitudinal identity and participation trajectories
Enable distributed expertise	Content coverage metrics	Interaction patterns and collective sense-making

### 5.2. Pedagogical Alignment and Assessment

For pedagogical alignment to operate coherently, learning objectives within ECHO must reflect the type and depth of learning the architecture is designed to support. Constructive alignment emphasizes coherence between intended learning outcomes, learning activities, and assessment approaches, with learning theories informing these design decisions [17,18]. Bloom’s taxonomy and its later revision classify learning objectives by increasing levels of cognitive engagement—from understanding and application to analysis, evaluation, and creation—providing a shared language to articulate the depth of learning expected from educational activities [31,32].

Within ECHO, objectives formulated at lower cognitive levels can address legitimate education goals, such as increasing awareness of emerging practices or reinforcing foundational clinical knowledge. These objectives often support the short didactic segments that precede case discussions in many ECHO sessions. At the same time, the model relies heavily on interactive case presentations and collaborative discussions, where clinicians collectively interpret clinical situations, exchange perspectives, and formulate practice recommendations. This case-based telementoring structure is widely described in the ECHO literature as a mechanism through which participants develop clinical knowledge, skills, and confidence in managing complex conditions in their own practice settings [11,33,34,35].

When ECHO programs seek to support these forms of collaborative clinical reasoning and practice adaptation, learning objectives that target higher-order cognitive processes, such as analysis, evaluation, or application in context, may align particularly well with the interactive learning architecture of the model. Framing objectives at these levels allows learning activities, participant interaction, and evaluation strategies to converge around the interpretative and decision-making processes that characterize case-based professional learning.

Examples of this alignment, alongside illustrative formulation of lower-level objectives, associated learning activities, and corresponding evaluation foci, are presented in Table 3.

**Table 3:** Pedagogical Alignment in ECHO: Objectives, Activities, and Evaluation.

Learning Focus	Architecture-Aligned ECHO Learning Objective	Bloom’s Cognitive Level (Definition)	ECHO Learning Activities Supporting the Objective	Example of Lower-Level Objective (Limited Alignment with ECHO Architecture)	Evaluation Focus Aligned with Learning Processes
Clinical reasoning	Analyze complex cases by integrating clinical evidence with patient and system constraints	Analyze (examine relationships); Evaluate (justify decisions)	Participant-presented cases; facilitated group reasoning; comparison of management options	Increase knowledge of clinical guidelines	Transparency and evolution of reasoning across cases
Practice adaptation	Justify and adapt clinical decisions to local practice environments	Evaluate (judge appropriateness); Create (generate adapted strategies)	Peer exchange; expert questioning; discussion of feasibility and constraints	Become familiar with recommended practices	Justification of adaptations and contextual decision-making
Collective inquiry	Contribute actively to shared problem-solving within the learning community	Apply (use knowledge in context); Analyze (consider multiple perspectives)	Longitudinal participation; questioning peers; responding to prior cases	Participate in ECHO sessions	Quality and diversity of contributions over time
Professional identity	Articulate professional judgment and assume responsibility within the learning community	Analyze (reflect on role); Evaluate (defend professional stance)	Case ownership; feedback exchange; reflective dialogue	Increase confidence in managing cases	Trajectories of participation, legitimacy, and role assumption
Metacognitive regulation	Reflect on uncertainty and adjust reasoning strategies over time	Evaluate (reflect critically); Create (modify strategies)	Facilitated reflection; revisiting earlier cases	Understand limits of clinical decision-making	Evidence of reflection and adaptive reasoning across sessions

The examples presented in Table 1, Table 2 and Table 3 are intended to support pedagogical and analytical reflection on learning processes within Project ECHO. Further empirical research is required to examine their validity, reliability, and applicability across context.

The following example illustrates how a program objective can be aligned with a case-based activity, facilitation strategies, observable learning indicators, and corresponding assessment approaches within an ECHO session.

For example, within the ECHO program on Infection-Associated Chronic Conditions (IACC) implemented at CHUM, one learning objective is to support the exchange of best practices and evidence-informed approaches for the management of patients living with chronic infectious conditions.

To support this objective, a session may include a participant-presented clinical case involving a patient living with HIV who also presents with multiple chronic comorbidities (see Table 4). During the discussion, facilitators may invite participants to explain the clinical reasoning underlying their proposed management strategies, compare alternative approaches, and discuss how guideline recommendations can be applied or adapted within different care contexts.

Within this interaction, several learning processes may become observable. Participants may share practice-based experiences, reference clinical evidence, and articulate the reasoning guiding their decisions. Over time, these exchanges may contribute to the development of a shared repertoire of practices and approaches within the group.

**Table 4:** Example of pedagogical alignment within an ECHO session (CHUM IACC program).

Program Objective	ECHO Learning Activity	Facilitation Strategies	Observable Learning Indicators	Possible Assessment Methods
Exchange evidence-informed practices for the management of patients living with infection-associated chronic conditions	Participant presents a complex clinical case involving a patient living with HIV and multiple chronic comorbidities	Facilitator invites participants to articulate the reasoning underlying their proposed management strategies; encourages comparison of alternative approaches; prompts discussion of contextual constraints affecting care	Participants reference clinical evidence during discussion; participants explain decision criteria guiding their proposed management strategies; participants compare alternative management options; participants acknowledge contextual factors such as resource availability or care coordination constraints	Structured facilitator observation of reasoning articulation and references to evidence; documentation of participation patterns and peer exchange; coding of discussion transcripts to identify reasoning structures and use of evidence

These learning processes can be examined through different assessment approaches. At a pragmatic level, facilitators may document participation patterns and references to evidence during discussions through structured observation. At a more analytically intensive level, recordings or transcripts of discussions may be analyzed to examine how participants mobilize clinical evidence and build on each other’s contributions during case-based dialogue.

## 6. Institutional Pedagogical Leadership

Building and sustaining a learning architecture such as Project ECHO requires more than pedagogical intent at the level of individual programs. Research in teaching and learning can provide a conceptual and methodological foundation for making learning processes visible, informing design choices, and supporting sustained alignment between educational objectives, learning activities, and assessment over time.

In the Quebec context—and more specifically within CHUM—the ECHO ecosystem illustrates how an institutional Directorate of Teaching and Learning can create the conditions to operationalize this role. By articulating learning principles, structuring faculty development around facilitation and learning processes, and embedding research-informed reflection into program design, the Direction de l’enseignement et de l’Académie (DEAC) establishes a governance structure that supports pedagogical coherence and the long-term sustainability of ECHO initiatives.

In practical terms, such pedagogical leadership may include facilitator development focused on questioning and dialogic moderation, review of learning objectives across programs, support for aligning assessment strategies session design, and structured reflection on how participation and reasoning evolve over time. In this sense, CHUM serves as practical example of how pedagogical leadership can support facilitator development, alignment of learning objectives with session design, and the identification of process-oriented approaches to evaluation. The CHUM experience is presented here as an illustrative institutional context rather than as an empirical case study.

Beyond a local case, this experience points to a transferable roadmap for institutions seeking to strengthen telementoring initiatives as learning systems. At CHUM, this roadmap begins by making pedagogical assumptions explicit through the use of learning theory to guide ECHO program design and the formulation of learning objectives that reflect the depth of learning expected. It then extends to faculty development, with a focus on facilitation practices that promote interaction, clinical reasoning, and collective sense-making rather than content transmission alone. Building on this alignment, insights from the learning sciences inform the identification of process-oriented indicators that correspond to these objectives and complement outcome-focused CPD frameworks.

When embedded within an institutional structure, these functions support continuity, reflexivity, and adaptation over time. Together, they provide a coherent basis for revisiting and strengthening the assessment structure of ECHO programs and establishing a conceptual and analytical foundation for future research in teaching and learning focused on examining how learning unfolds within ECHO, rather than reporting empirical findings at this stage.

## 7. Discussion and Conclusions

Reframing ECHO as a learning architecture rather than solely as a telementoring delivery model offers a complementary perspective to the existing literature on digital continuing professional development (CPD). Reviews of ECHO evaluations have emphasized organizational reach, participation, and downstream clinical or service-level outcomes, often using outcome-oriented structures to organize reported results [5,6,7]. These approaches support accountability and comparability across programs, but they offer limited insight into how professional learning develops during participation in telementoring interactions [5,7,14].

The present analysis addresses this limitation by applying learning-theory perspectives to clarify the pedagogical processes through which learning can develop within Project ECHO sessions. Situated learning, communities of practice, and cognitive apprenticeship together highlight learning as a socially mediated process that develops through engagement with authentic clinical cases, participation in collective reasoning, and guided articulation of expert thinking [26,27,28]. Within this perspective, learning emerges through interaction: participants contribute practice-based experience, compare interpretations of clinical problems, and progressively refine their reasoning through dialogue with peers and specialists.

From this theoretical standpoint, the widely cited principle of “all teach, all learn” can be interpreted as a structured form of distributed expertise within a learning community [36]. The effectiveness of this learning structure depends on pedagogical conditions that support meaningful participation, including the relevance of presented cases, facilitation practices that prompt explanation and comparison of clinical reasoning, and interactional dynamics that enable legitimate participation among members of the group [2,26,27,28]. These conditions align closely with the mechanisms described in community of practice and cognitive apprenticeship, where learning occurs through participation in shared problem-solving and through the progressive externalization of expert reasoning.

This process-oriented analysis also clarifies the relationship between pedagogical design and CPD evaluation. Outcome frameworks such as Moore’s model remain central in CPD for organizing evidence across levels of impact, from participation and satisfaction through learning, competence, and, in some cases, performance and patient outcomes [14,15]. Reviews of ECHO evaluations have explicitly used this framework to organize reported outcomes, particularly at the levels of participation, learning, and self-reported competence [5,7].

This perspective also highlights the importance of pedagogical alignment in ECHO program design. Constructive alignment [37] emphasizes coherence between intended learning objectives, learning activities, and assessment approaches [17,18]. Within the ECHO architecture, case-based discussions serve as the primary learning activity through which participants engage with authentic clinical uncertainty. Facilitation practices guide participants in articulating reasoning, examining alternative management strategies, and connecting evidence with local practice constraints. Assessment approaches must therefore attend not only to outcomes of participation but also to the interactional processes through which reasoning and shared repertories develop.

Within this broader perspective, outcome frameworks sch as Moore’s model remain useful for organizing evidence of program impact in CPD contexts [14,15]. However, these frameworks primarily structure evaluation after participation and not specify the pedagogical mechanisms through which learning unfolds during collaborative discussion. By contrast, the framework proposed in this article clarifies how learning processes can be examined within Program ECHO sessions themselves by focusing on participation dynamics, articulation of clinical reasoning, and development of shared practice through dialogue.

The CHUM ECHO ecosystem illustrates how institutional pedagogical leadership can support these principles across programs. A Directorate of Teaching and Learning can promote coherence by articulating shared learning principles, supporting faculty development focused on facilitation practices that encourage reasoning and dialogue, and aligning program objectives, activities, and evaluation approaches over time [2,17,18,26,27,28]. In this way, institutional structures can help sustain ECHO as a learning architecture rather than as a series of independent telementoring initiatives.

In conclusion, Project ECHO represents more than a technology-enabled mechanism for extending specialist expertise; it constitutes a learning architecture that can support meaningful professional learning when programs design, facilitation practices, and evaluation strategies are aligned with learning theories. By clarifying the pedagogical mechanisms that structure interaction within ECHO sessions, this framework complements existing outcome-oriented CPD evaluations and provides a conceptual foundation for future research examining how learning develops within digital collaborative networks [14,15,16,17,18,26,27,28].

## 8. Limitations

This article develops a conceptual analysis and does not present empirical data. Several limitations therefore require consideration.

First, the proposed framework has not yet undergone empirical testing within Project ECHO programs. Empirical studies will need to examine how the proposed constructs operate in authentic program contexts. Second, the theoretical perspectives used in this analysis represent a limited selection of frameworks that address professional learning in digital environments. Other theoretical approaches could enrich the interpretation of learning processes in ECHO and provide additional analytical lenses. Third, the operational indicators presented in the tables remain conceptual. Researchers must evaluate their feasibility, clarity, and reliability when applied to real ECHO activities and data sources.

Transferability of the framework should also be considered in relation to program maturity, facilitation capacity, technological infrastructure, participant continuity, and the degree to which interactional learning is explicitly supported as a pedagogical goal. Future research should examine how the proposed learning processes appear during actual ECHO interactions and should test whether process-oriented indicators strengthen the evaluation of learning in continuing professional development. Such work could also clarify how process-based analysis complements established outcome-focused CPD evaluation models.

## Data Availability

No new data were created or analyzed in this study. Data sharing is not applicable to this article.
